# The Roles of Mitochondrion in Intergenomic Gene Transfer in Plants: A Source and a Pool

**DOI:** 10.3390/ijms19020547

**Published:** 2018-02-11

**Authors:** Nan Zhao, Yumei Wang, Jinping Hua

**Affiliations:** 1 Laboratory of Cotton Genetics, Genomics and Breeding/Key Laboratory of Crop Heterosis and Utilization of Ministry of Education, College of Agronomy and Biotechnology , China Agricultural University, Beijing 100193, China; Nan_Zhao@cau.edu.cn; 2 Institute of Cash Crops, Hubei Academy of Agricultural Sciences, Wuhan 430064, China; yumeiwang001@126.com

**Keywords:** mitochondrion, intergenomic gene transfer, nucleus, chloroplast, genome evolution

## Abstract

Intergenomic gene transfer (IGT) is continuous in the evolutionary history of plants. In this field, most studies concentrate on a few related species. Here, we look at IGT from a broader evolutionary perspective, using 24 plants. We discover many IGT events by assessing the data from nuclear, mitochondrial and chloroplast genomes. Thus, we summarize the two roles of the mitochondrion: a source and a pool. That is, the mitochondrion gives massive sequences and integrates nuclear transposons and chloroplast tRNA genes. Though the directions are opposite, lots of likenesses emerge. First, mitochondrial gene transfer is pervasive in all 24 plants. Second, gene transfer is a single event of certain shared ancestors during evolutionary divergence. Third, sequence features of homologies vary for different purposes in the donor and recipient genomes. Finally, small repeats (or micro-homologies) contribute to gene transfer by mediating recombination in the recipient genome.

## 1. Introduction

A billion years ago, a host cell engulfed a dependent bacteria, α-proteobacteria, which turned into a semi-autonomic organelle, a mitochondrion [1]. It delivers energy to the eukaryotic host cell in the diversifying evolution [2]. Meanwhile, mitochondrial sequences transferred among intracellular genomes [3,4,5,6,7], which is intergenomic gene transfer (IGT). On the one hand, nuclear transposons and chloroplast tRNA genes transferred into the mitochondrial genomes in most seed plants [5,7,8,9,10,11,12]. Nuclear sequences contributed to mitogenome expansion, contributing almost half in melons [10]. Among the nuclear-like sequences in the mitochondrial genome, the long terminal repeat retrotransposons (LTR-retro) ranked first [4,7,8,9,10,11,12]. In addition, chloroplast-like genes promoted the translation in mitochondrial genome [13,14,15]. The sequence states of chloroplast genes changed in the donor (chloroplast) and receptor (mitochondrion) genomes [16,17], which concerned their later roles [18]. Besides, DNA sequence microhomology played an important role in chloroplast DNA inserting into the mitochondrion, which might be the microhomology-mediated break-induced replication (MMBIR) [19] or non-homologous end joining (NHEJ) [20].

On the other hand, a large-scale of mitochondrial genes moved into the nucleus and chloroplast [21,22,23,24,25]. The prokaryotic genes (mitochondrial genes) converted to eukaryotic genes (nuclear genes) [26] to engage in sexual recombination [27]. Besides, RNA could mediate mitochondrion-to-nucleus transfers [28,29]. The mitochondrial genes preferentially inserted in the open nuclear chromosome regions [30]. These nuclear integrants of mitochondrial genes (*numts*) would gradually decay or transform to nuclear sequences [31]. A few *numts* received nuclear promoters and transit peptides [2,32] that guided their products to the mitochondrion [33,34]. Few nuclear homologies of organellar DNA could transcribe successfully [35]. However, mitochondrion-to-chloroplast transfer only occurred in a few angiosperms [16,17,18,36,37,38,39,40,41]. Perhaps because plastids were conservative [17,36] and lacked efficient DNA uptake setups [42]. During the evolution, mitochondrial sequences moved to the chloroplast genomes of the shared ancestors of certain relative species [17,18,38,39]. They preferentially inserted into the intergenic spacer [16,17,41] or the large single copy (LSC) region of the chloroplast genomes [38]. The insertion accompanied DNA repair by homologous recombination [17]. However, most chloroplast homologies of mitochondrial genes had low transcriptional levels [17]. Environmental stresses could promote chloroplast [43] and nucleus [44] to absorb exogenous DNA. Meanwhile, the loss of mitochondrial membrane proteins could facilitate the export of the mitochondrial genes [2].

Recently, rapid development of genomic sequencing technologies has made it feasible to approach more IGT events in plants. It enables us to look into the details of intergenomic gene transfer. In this paper, we unveil the IGT events related to the mitochondrion based on 24 sets of nuclear, mitochondrial and chloroplast genomic sequences in plants (Table S1). We expect these results will lay the foundation for further exploration of genome evolution.

## 2. Results and Discussion

### 2.1. The Role of Mitochondrion as a Gene Source: Intergenomic Gene Transfer from Mitochondrion

#### 2.1.1. Intergenomic Gene Transfer from Mitochondrion to Nucleus

**Figure 1:**
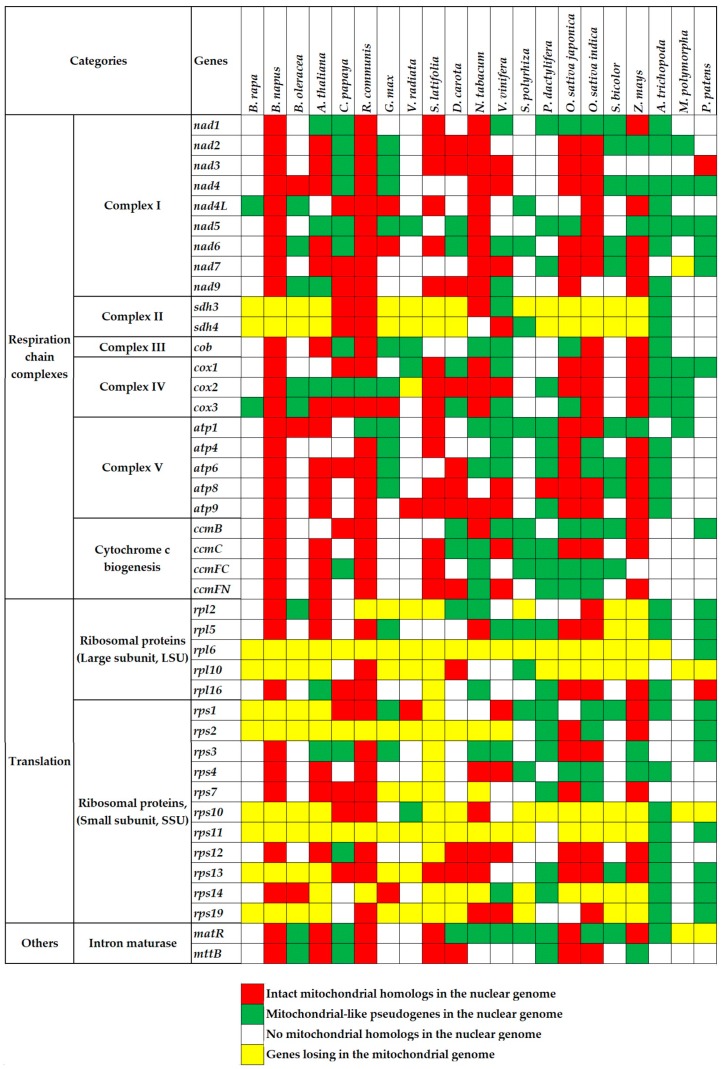
Genes identified to transfer in and out of the mitochondrial genome or genes lost from the mitochondrial genome of 21 land plants. The first two columns are mitochondrial protein-encoding genes (the second column) and their functional categories (the first column). The first line lists the names of plant species. The red and green cells represent mitochondrial full-length intact homologs and pseudogenes in nuclear genomes, respectively. The white and yellow cells represent no mitochondrial homologs in nuclear genomes and genes lost from mitochondrial genomes, respectively.

There exist a number of conserved genes during the mitochondrial genome evolution [45,46]. In the present study, we use 67 essential genes to study the gene loss and transfer about the mitochondrial genome. As a result, genes encoding complex II and ribosomal subunits have been lost massively in most of the higher plants (Figure 1, yellow cells). Genes encoding complexes III and V display much greater conservation. These gene losses are parts of the mitochondrial genome variations in plants. Our next goal is to elucidate where the lost genes transferred. Two of the main detectable destinations are nuclear and chloroplast genomes.

Transferred genes exist in two forms: remnants left in the mitochondrial genome [47] and fragments inserted into the nuclear genome (*numts*) [48,49]. Few *numts*’ products returned to the mitochondrion and played a role [33,34]. Researchers have achieved the mitochondrion-to-nucleus transfer by experiments, whose flow was as follows: (1) introduce a silent selectable marker gene with a nuclear promoter and transit peptide-encoding sequence into the mitochondrial genome; (2) transform this recombinant mitochondrion into a new cell; (3) detect the phenotype related to the marker gene. This approach has been successful in the unicellular green alga *Chlamydomonas reinhardtii* [27]. However, there is no experimental report on the real mitochondrion-to-nucleus IGT. Mitochondrial genes transferred with prokaryotic signals, which needed a long time or a favorable evolutionary event to turn into the eukaryotic ones.

**Figure 2:**
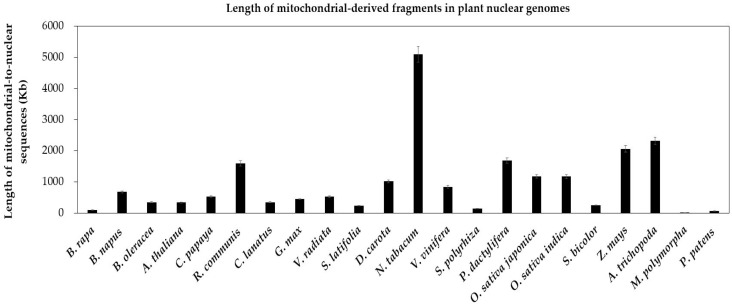
Length of mitochondrial-derived fragments in plant nuclear genomes. The plant species are arrayed on the horizontal axis. The total lengths of mitochondrial-to-nuclear sequences are along the vertical axis. The bars represent the lengths of sequences transferring from the mitochondrion to the nucleus in plant species. The error bars stand for the positive and negative deviations of 5.0%.

In present research, to identify possible *numts*, we carry on non-experimental analyses by performing the genome alignment between conserved mitochondrial genes and nuclear genomes in above 21 land plants. First, we find extensive gene transfer and gene loss in these plants (Figure 1). Second, the gene transfer is more popular in eudicots and monocots than that in bryophytes. Specifically, the latter is merely 1/20 of the former (Figure 2). Since the bryophytes with few mitochondria could not survive after vast transfer [24]. Third, we identify a number of full-length mitochondrial-like protein-coding genes in the nuclear genome (Figure 1, red cells), which may be useful candidate genes. Fourth, there are also mitochondrion-like truncated genes, which we define as pseudogenes (Figure 1, green cells).

Genes integrated by nuclear genome have different endings. Nearly all lost their original roles and became a part of new nuclear sequences [31]. A few could re-gain function by receiving nuclear promoter and transit peptide [2,32]. Others would suffer from irreversible decay with accumulating an increasing number of unfavorable mutations. These events allow prokaryotic gene(s) to turn into eukaryotic gene(s) [26] to join in sexual recombination [27]. As for the transferred forms, early studies displayed RNA-mediated gene transfers from the mitochondrion to the nucleus [28,29], while DNA-mediated gene transfer was rare in plants. In addition, the lack of integral mitochondrial membrane proteins could hasten the gene export from the mitochondrion [2].

**Figure 3:**
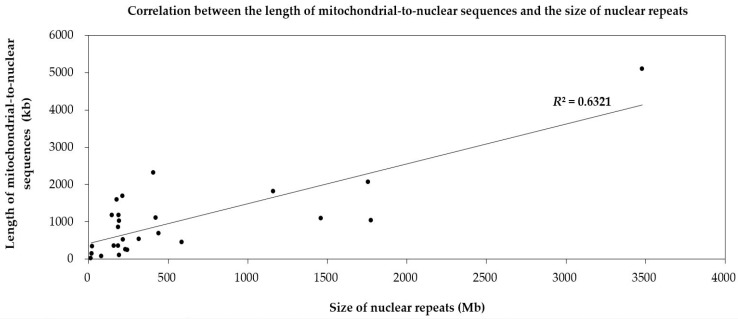
Correlation between the length of mitochondrial sequences transferring to the nucleus and repeat sizes of the nuclear genome in 22 land plants. Each dot represents a length value (X, Y). X refers to the size of the repeats in nuclear genomes of one species (based on the horizontal axis). Y means the length of mitochondrial-to-nuclear sequences in its corresponding species (based on the vertical axis). The slash represents the linear regression function of the distribution tendency of the dots. *R*^2^ is the regression coefficient.

To dissect the mechanism of mitochondrion-to-nucleus gene transfers, we analyze the repeats in nuclear genomes of 22 land plants. The ratios of the repeat size to the genome size of the four species, including two bryophytes (*M. polymorpha* and *P. patens*) and two angiosperms (*A. thaliana* and *S. polyrhiza*), are less than 20% (Table 1). Meanwhile, these four species contain fewer *numts* than other species (Figure 1). And there is a positive correlation between *numts* and the repeats in the nuclear genome (*R*^2^ = 0.6321) (Figure 3). The weak correlation may due to limited number of plant species used in present research. So, we consider that *numts* may become parts of the nuclear repeats to take part in repeat-mediated sexual recombination for a greater genetic diversity.

#### 2.1.2. Intergenomic Gene Transfer from Mitochondrion to Chloroplast

Given the prevailing mitochondrion-to-nucleus IGT, similar transfers into the chloroplast might be expected. However, mitochondrion-to-chloroplast IGT happened only in three angiosperms, *Apiaceae* [16,18,36,37,38,39], *Apocynaceae* [17] and *Poaceae* [40,41]. The first two families belong to the eudicots and the last to the monocots.

The existing forms of gene sequences in and out of both donor and receptor genomes altered after the transfer. *D. carota* Mitochondrial Plastid sequence (*Dc*MP)—presented three fragment sequences (*Dc*MP 1, −2 and −3 +4) in the plastid genome. The split probably arose from new DNA recombination that happened after one copy of *Dc*MP migrated into the mitochondrial genome [16]. Besides, mitochondrial-like *rpl2* only contained an exon in the plastid genome and two homologies in different regions of the mitochondrial genome in *A. syriaca* [17]. In addition, the traits of gene sequences in the plastid genome (recipient genomes) might affect their specialized roles. *Dc*MP inserted into two short direct repeats in the plastid genome, which suggested that it served as non-LTR retrotransposon [18]. For those mitochondrial-derived pseudogenes in the plastid, they contained nonsense mutations that would lead to a premature stop codon, which was consistent with the low transcriptional level of the plastid copy *rpl2* in *A. syriaca* [17].

From an evolutionary perspective, mitochondrion-to-chloroplast transfer occurred in the earlier common ancestor of certain relative species as a single event. For example, the homolog of mitochondrial gene, *Dc*MP, existed in the plastid genomes of *Daucus* and their close relative *Cuminum* [18]. Further studies showed that *Dc*MP moved to the shared ancestor of *Daucinae Dumort* and *Torilidinae Dumort* subtribes after they diverged from their ancestral tribe, *Scandiceae Spreng* [38,39]. Also, in *Apocynaceae*, mitochondrial *rpl2* transferred to the plastid genome of the common ancestor of the *Asclepiadeae* and *Eustegia* [17].

Mitochondrial sequences preferentially inserted into the intergenic spacer of plastid genomes. For instance, *Dc*MP inserted in the *rps12-trnV* intergenic spacer in the *D. carota* plastid genome [16]. There were also mitochondrial insertions in the *rps2-rpoC2* intergenic spacer of the plastid genome in *A. syriaca* [17] and in the *rpl23-ndhB* intergenic spacer of the plastid genome of *Parianinae* (*Eremitis* sp. and *Pariana radiciflora*) [41]. Besides, another mitochondrial-to-nuclear transfer appeared in the large single copy (LSC) region between the junction with inverted repeat A (IRA) and tRNA-His (GUG) (*trnH*-GUG) in limited *Apiaceae* species [38]. Additionally, insertion locations implied the roles of the transferred genes. *Dc*MP was regarded as a non-LTR retrotransposon targeting tRNA-coding regions because it moved to the upstream of the *trnV* gene in the plastid genome. Otherwise, *Dc*MP worked as three new promoters (P1–P3) that substituted two original promoters of the *trnV* gene (P4 and P5) [18]. More importantly, insertion typically came with DNA repair of a double-stranded break by homologous recombination. To create homologies, the plastid gene *rpoC2* preferentially inserted into the mitochondrial genome, just near the mitochondrial-native gene *rpl2*, then intact mitochondrial *rpl2* and part of *rpoC2* transferred together to the plastid of *A. syriaca* [17].

### 2.2. The Role of Mitochondrion as a Gene Pool: Intergenomic Gene Transfer into Mitochondrion

#### 2.2.1. Intergenomic Gene Transfer from Nucleus to Mitochondrion

**Figure 4:**
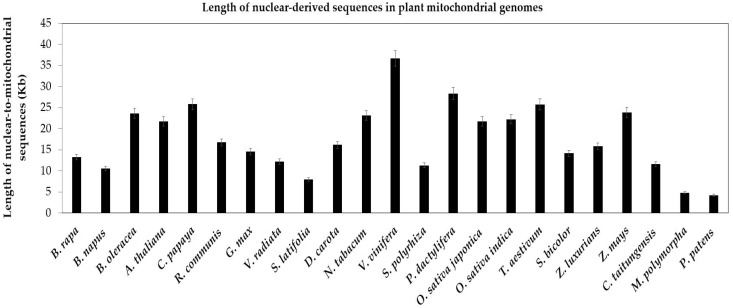
Length of nuclear-derived sequences in plant mitochondrial genomes. The plant species are arrayed on the horizontal axis. The total lengths of nuclear-to-mitochondrial sequences are along the vertical axis. The bars represent the lengths of sequences transferring from the nucleus to the mitochondrion in plant species. The error bars stand for the positive and negative deviations of 5.0%.

Compared with the conservative chloroplast genome, the mitochondrial genome diversified among plant species. The primary drivers of genome variations might be repetitive sequences and nuclear-derived DNA, which represented 42% and 47% of the total sequences in melon, respectively [10]. In present study, we analyze the nucleus-to-mitochondrion sequences of 23 plants. First, nuclear-derived sequences are widespread in all mitochondrial genomes of 23 plants (Figure 4). Second, among spermatophytes, total nuclear sequences in mitochondrial genomes range from a low of 7960 bp in *S. latifolia* to a high of 36,123 bp in *V. vinifera* (Table S2). Third, the nucleus-to-mitochondrion transferred sequences are less in bryophytes than in spermatophytes, 4249 bp and 4814 bp in *P. patens* and *M. polymorpha*, respectively (Figure 4).

**Figure 5:**
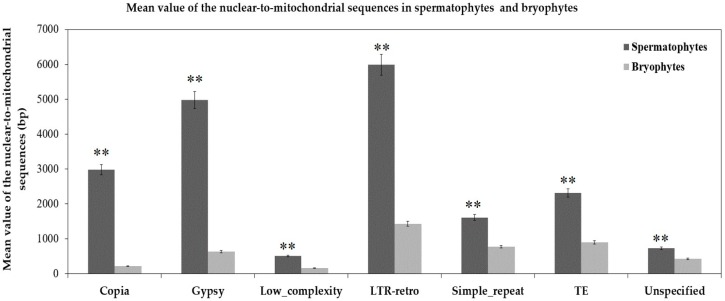
Mean value of the length of different nuclear sequences transferring to the mitochondrial genomes of spermatophytes and bryophytes. ** *p* < 0.01. The seven categories of repeats are arrayed on the horizontal axis. The total lengths of nuclear-to-mitochondrial repetitive sequences are along the vertical axis. The dark gray and light gray bars represent the mean values of repeats transferring from the nucleus to the mitochondrion in 21 spermatophytes and 2 bryophytes, respectively. The error bars stand for the positive and negative deviations of 5.0%.

**Figure 6:**
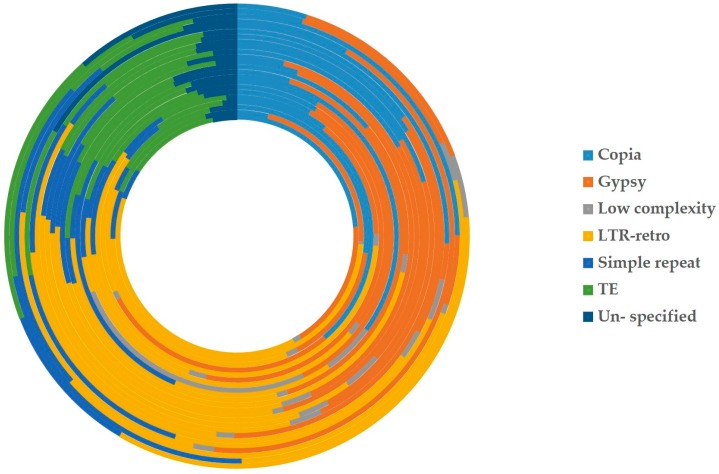
Percentages of each kind of repeats from all nuclear-to-mitochondrial repetitive sequences in 23 plants. The 23 circles represent the whole nuclear-to-mitochondrial repeats of 23 plants inside and out. The boxes in different colors on the right are the symbols of seven kinds of repetitive sequences. (From top to bottom) Light blue: copia; Orange: gypsy; Gray: low complex; Yellow: LTR-retro (long terminal repeat retrotransposons); Middle blue: simple repeat; Green: TE (transposable element); dark blue: un-specific.

**Figure 7:**
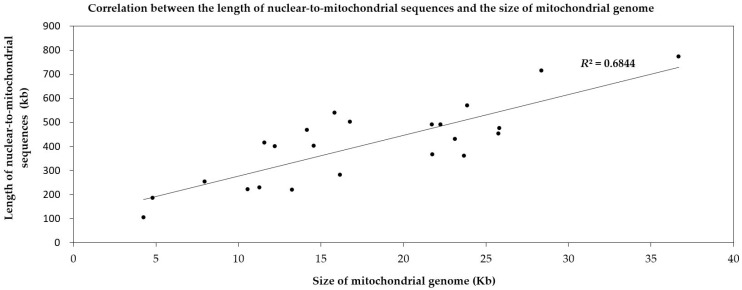
Correlation between the length of nuclear sequences transferring to the mitochondrion and the size of the mitochondrial genome in 23 land plants. Each dot represents a length value (X, Y). X refers to the length of the mitochondrial genome of one species (based on the horizontal axis). Y means the length of nuclear-to-mitochondrial sequences in this corresponding species (based on the vertical axis). The slash represents the linear regression function of the distribution tendency of the dots. *R*^2^ is the regression coefficient.

According to the different degrees of the matching and annotation, these nuclear-to-mitochondrial repetitive sequences fall into seven categories: copia, gypsy, low complexity, long terminal repeat retrotransposons (LTR-retro), simple repeat, transposable element (TE) and unspecified (Table S2). Copia and gypsy represent two main classes of LTR-retrotransposons that belong to Class 1 transposable elements [72]. Low-complexity DNA primarily include poly-purine/poly-pyrimidine stretches and regions of extremely high AT or GC content. First, the mean of each type in 21 spermatophytes is significantly larger than that in 2 bryophytes (Figure 5), which show most nucleus-to-mitochondrion transfers occurred after the differentiation of seed plants and bryophytes, at least, for the analyzed 2 bryophytes species. Second, the first three are LTR-retro, gypsy and copia in 23 plants (Figure 6, Table S2). This result conforms to the early discoveries in a number of plants, including the gymnosperm *Cycas taitungensis* [9], the monocot *Oryza sativa* [8] and the eudicots *Arabidopsis thaliana*, *Cucumis melo* and *Cucumis sativus* [4,7,10,11,12]. Third, the total length of transferred sequences correlates with the mitogenome size (Figure 7). This result supports the import of promiscuous DNA is a core mechanism for mitochondrial genome expansion in land plants [73].

#### 2.2.2. Intergenomic Gene Transfer from Chloroplast to Mitochondrion

**Figure 8:**
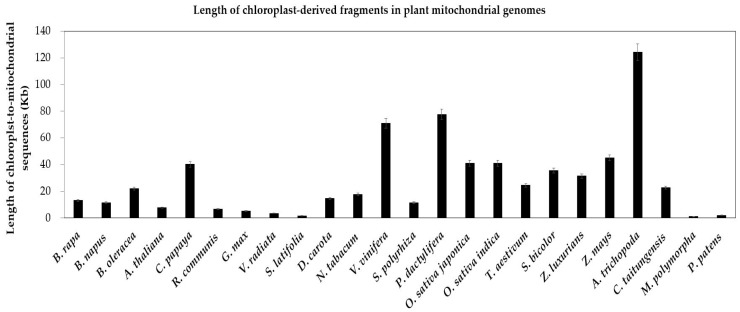
Length of chloroplast-derived fragments in plant mitochondrial genomes. The plant species are arrayed on the horizontal axis. The total lengths of chloroplast-to-mitochondrial sequences are along the vertical axis. The bars represent the lengths of sequences transferring from the chloroplast to the mitochondrion in species. The error bars stand for the positive and negative deviations of 5.0%.

As with mitochondrial genomes, chloroplast genomes also contain a minimum set of largely conserved protein-encoding, rRNA and tRNA genes [21,74,75]. In contrast to the extensive gene loss of mitochondrial genomes, only few chloroplast-encoded genes have been lost in chloroplast genomes of specific plants (Figure S1, yellow cells). For example, three genes (*accD*, *ycf1* and *ycf2*) are lost in the grasses (*O. sativa japonica*, *O. sativa indica*, *S. bicolor*, *Z. mays*), another three genes (*ccsA*, *rpoA* and *rpl16*) are lost in the moss *P. patens* (Figure S1, yellow cells). Compared to a few gene loss, chloroplast genes transferring to nucleus and mitochondrion are richer (Figures S1 and S2). In our study, we unearth the enormous chloroplast-to-mitochondrion gene transfers in 24 land plants. Similar gene copies exist in two contemporary intracellular genomes simultaneously (Figure S1, the red and green cells). In two bryophytes, the total lengths of integrated sequences are close, 1.05 kb in *M. polymorpha* and 1.99 kb in *P. patens* (Figure 8). In addition, the variation range is greater in 22 seed plants, from 1.67 kb in *S. latifolia* to 130 kb in *A. trichopoda* (Figure 8). Besides, the chloroplast-to-mitochondrion fragments of most seed plants are more than that in bryophytes (Figure 8).

Large parts of chloroplast tRNA genes immigrated into plant mitochondrial genomes [5,9]. These transfers were essential to the translation of the mitochondrial genes [13,14,15]. Here, we identify the chloroplast-like tRNA genes in the mitochondrial genome of 24 plants species using blast. And then we build a phylogenetic tree to elucidate the evolutionary implications. First, there is no chloroplast-derived tRNA gene in mitochondrial genomes of two bryophytes (Figure S2). Second, single or multiple chloroplast genes immigrated to the mitochondrial genomes of spermatophytes, at least, for the analyzed 21 angiosperms and 1 gymnosperms. For example, (1) chloroplast-like *trnM* gene appears in the mitochondrial genomes of all studied seed plants except *Z. mays*, which suggests that chloroplast *trnM* lost only in *Z. mays* during or after transferring to the mitochondrion and this transfer happened with spermatophytes and bryophytes diverging; (2) chloroplast *trnH* gene transferred to the mitochondrial genomes of most spermatophytes but lost in *P. dactylifera*, *T. aestivum* and *G. biloba*, which might be the random loss; (3) *trnN*, *trnP*, *trnS* and *trnW* transferred merely in angiosperms, despite parts of these four genes lost in a few species; (4) chloroplast *trnD* gene moved into the mitochondrion only in eudicots, which shows that *trnD* transferred when eudicots and monocots diverged; (5) chloroplast-like *trnC* gene and *trnF* gene transferred to the mitochondrion simply in *Gramineae* crops of monocots; (6) ten chloroplast-to-mitochondrion genes (*trnD*, *trnE*, *trnG*, *trnI*, *trnK*, *trnL*, *trnP*, *trnR*, *trnT* and *trnY*) transferred together in *V. vinifera* (Figure S2).

**Figure 9:**
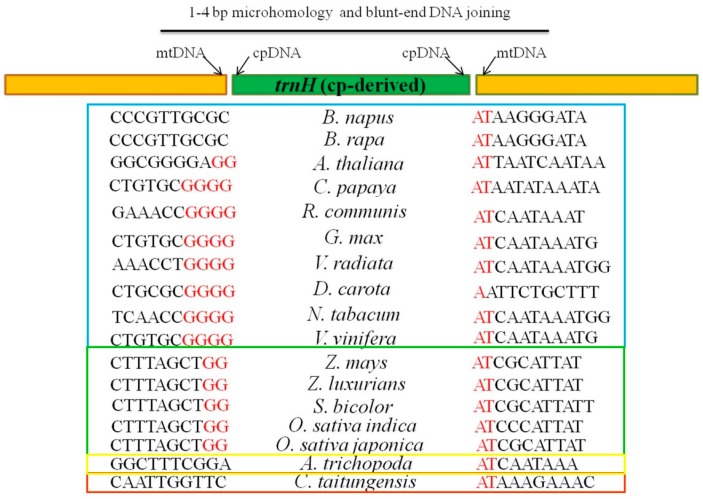
Nucleotide-resolution analysis on flanking sequences of the chloroplast-derived *trnH* gene in mitochondrial genomes of most spermatophytes. cpDNA and mtDNA are the abbreviations of chloroplast DNA and mitochondrial DNA. The yellow-green-yellow strip represents the fusion sequence of mtDNA-cpDNA-mtDNA. The sequences under the two yellow strips on the left and right are the flanking sequences of inserted chloroplast-like tRNA gene in the mitochondrial genomes. The red capital English letters close to cpDNA indicate the nucleotides of micro-homologies among the different species. The species in the blue, green, yellow and red boxes belong to eudicots, monocots, basal angiosperms and gymnosperms, respectively.

To infer the mechanism of chloroplast tRNA genes inserting into mitochondria, we analyze the flanking nucleotide sequences in insertion sites of mitochondrial genomes. *trnH* transferred in most spermatophytes (Figure 9). *trnD* moved specifically in eudicots (Figure S3). *trnC* and *trnF* migrated only in *Gramineae* crops (Figure S4). Taking together, we notice the micro-homologies (1 to 4 bp) among plant species in the breakpoint sequences of chloroplast-mitochondrial DNA fusion. The micro-homologies are the same adenine-thymine (AT) on the right of *trnH* in spermatophytes. But on the left are four short tandems Guanine (G) in eudicots, two repeated Guanine (G) in monocots and no microhomology in gymnosperms (Figure 9). Therefore, we confer that DNA sequence microhomology plays an important role in chloroplast DNA inserting into the mitochondrion, which may be the microhomology-mediated break-induced replication (MMBIR) [19] or non-homologous end joining (NHEJ) [20].

On top of it all, we infer the repeats in mitochondrial genomes have the potential to mediate DNA recombination, which contributes to gene transfer and reuse of the transferred genes in target genomes. Therefore, we analyze the repeats variation in recipient genomes (the mitochondrial genomes) of land plants (Table 2) to explain various rates of gene transfer to some extent. First, plants with smaller values of repeat size, repeat number (>1 kb) and repeat number (>100 bp) contain less gene transfer, among which the most obvious is a bryophyte *P. patens* (Table 2 and Figure 8). Second, small repeats (>100 bp) are more favorable to gene transfer than large repeats (>1 kb) (Table 2).

## 3. Materials and Methods

### 3.1. Availability of Chloroplast, Mitochondrial and Nuclear Genomes

We download all the chloroplast, mitochondrial and nuclear genome sequences and gene annotations from NCBI database. And then we list all the accession numbers in Table S1.

### 3.2. Detection of Total Intergenomic-Transfer DNA Sequences

For 24 land plants, we align the sequences of chloroplast and mitochondrial genomes to nuclear chromosomes to detect nuclear insertions of chloroplast DNA (*nupts*) and nuclear insertions of mitochondrial DNA (*numts*) using the BLAST program. We set *e*-value to 1e^−5^ [76]. The minimum length of an exact match (95%) is 100 bp. While identifying mitochondrial insertions of chloroplast DNAs (*mtpts*) by local BLASTN (version 2.2.23) [76], we set the minimum length of an exact match to be 50-bp.

### 3.3. Identification of Intergenomic-Transfer Homologies

Taking a set of essential chloroplast or mitochondrial genes as references (Table S3), we gain their copies in the donor and recipient genomes using the BLAST program with the same parameters above [76]. If there is no counterpart in the donor genomes (chloroplast or mitochondrial genomes), we would consider them as the lost genes (Figure 1 and Figure S1, the yellow cells). To those presented in the donor genomes but absent in the recipient genomes, we consider that they did not transfer between two genomes (Figure 1 and Figure S1, the white cells). For those appearing concurrently in both donor and recipient genomes, we consider that their copies moved into another genome after duplication in the original genome (Figure 1 and Figure S1, the red and green cells). Further, we define the full-length copies of the transferred genes in the recipient genomes as the intact homologies (Figure 1 and Figure S1, the red cells). Otherwise, we recognize the truncated copies as pseudogenes (Figure 1 and Figure S1, the green cells). 

### 3.4. Detection of the Repeats in Mitochondrial Genomes

We detect nuclear-derived repetitive transposons using online software RepeatMasker (http://www.repeatmasker.org) in 24 land species and a custom repeats database. And then we use two-tailed *t*-tests to evaluate the significant difference of repeats between spermatophytes and bryophytes.

### 3.5. NHEJ Analysis

We perform the NHEJ analysis as previously described [77,78]. In short, *nupts*, *numts* or *mtpts* are inserted by NHEJ, like micro-homology or blunt end repair. If nucleotides close to the fusion point are similar in different land species, we would regard them as micro-homology. Otherwise, we would consider no micro-homology as blunt-end repair.

### 3.6. Phylogenetic Analysis

The phylogenetic analysis involves nucleotide sequences of 17 mitochondrial genes (*nad1*–*nad6*, *nad9*, *cob*, *cox1*–*cox3*, *atp1*, *atp4*, *atp6*, *atp8* and *atp9)*. We use the maximum likelihood (ML) method with the model GTR + G + I in MEGA5.05 [79]. And then we perform phylogenetic analyses according to the same methods in previous studies [80,81].

## 4. Conclusions

With the rapid development of genomic sequencing technologies, nuclear and organellar genomes data became available for many plants. Here, based on 24 sets of genome data, we detect and analyze intergenomic gene transfers (IGT) related to the mitochondrion. Meanwhile, we review the research advances of intergenomic gene transfer. As a summary, we find mitochondrion mainly plays two essential roles in gene transfer: Source and pool. From the source perspective, massive mitochondrial genes transfer into nuclear and chloroplast genomes. For the role of the pool, the mitochondrion integrates enormous genes from the other two genomes. Except for the disparate orientation, a lot of likenesses emerge when bringing them together. First, gene transfer related to mitochondrial genomes is prevalent in plants, though few genes flow from the mitochondrion to the chloroplast. Second, specific IGT is a single event of certain shared ancestors, which is consistent with the divergence clade. Third, an intact gene usually changes existing forms after transferring in and out of both donor and recipient genomes, which agrees with their consequent roles, such as, functioning like before, reusing for new loci or decaying gradually. Fourth, most exogenous DNA preferentially inserts into the intergenic region. Besides, small repeats (or micro-homologies) may contribute to gene transfers by mediating recombination in the recipient genomes. In a word, mitochondrial gene transfers dedicate to the genome variation and evolutionary diversity.

## Figures and Tables

**Table 1:** Variation of repeats in nuclear genomes of 22 land plants.

Species		Repeat Sizes (Mb)	Genome Sizes (Mb)	Repeat/Genome (%)	References
**Spermatophytes**	**Eudicots**				
	*B. rapa*	191.63	284.13	67.44	[50]
	*B. napus*	441.77	930.51	47.48	[51]
	*B. oleracea*	185.43	539.91	34.34	[52]
	*A. thaliana* ^1^	23.58	119.67	19.70	[53]
	*C. papaya*	316.53	369.78	85.60	[54]
	*R. communis*	176.00	350.62	50.20	[55]
	*C. lanatus*	159.80	321.05	49.77	[56]
	*G. max*	587.10	978.97	59.97	[57]
	*V. radiata*	216.17	548.08	39.44	[58]
	*S. latifolia*	244.82	665.28	36.80	[59]
	*D. carota*	193.70	473.00	40.95	[60]
	*N. tabacum*	3479.49	4500.00	77.32	[61]
	*V. vinifera*	185.35	487.00	38.06	[62]
	**Monocots**				
	*S. polyrhiza* ^1^	19.43	132.01	14.72	[63]
	*P. dactylifera*	214.34	558.02	38.41	[64]
	*O. sativa japonica*	188.00	374.42	50.21	[65]
	*O. sativa indica*	148.14	374.25	39.58	[66]
	*S. bicolor*	231.28	739.15	31.29	[67]
	*Z. mays*	1757.48	2067.62	85.00	[68]
	**Basal Angiosperms**				
	*A. trichopoda*	407.43	706.50	57.67	[69]
**Bryophytes**	*M. polymorpha* ^1^	12.48	304.37	4.10	[70]
	*P. patens* ^1^	79.37	477.95	16.61	[71]

^1^ notes repeat content less than 20%.

**Table 2:** Variation of repeats in mitochondrial genomes from 22 land plants.

Species		Mitochondrial Genome		
		Repeat Size (Kb)	Repeat Number (>1 kb)	Repeat Number (>100 bp)
**Spermatophytes**	**Eudicots**			
	*B. rapa*	3.80	1	9
	*B. napus*	4.62	1	17
	*B. oleracea*	152.00	2	24
	*A. thaliana*	15.63	2	25
	*C. papaya*	13.43	1	13
	*R. communis*	5.80	6	6
	*G. max*	60.67	13	68
	*V. radiata*	1.02	0	6
	*S. latifolia*	23.27	15	17
	*D. carota*	71.09	4	19
	*N. tabacum*	42.07	3	22
	*V. vinifera*	5.77	0	26
	**Monocots**			
	*S. polyrhiza*	1.58	0	5
	*P. dactylifera*	3.03	1	12
	*O. sativa japonica*	141.19	12	39
	*O. sativa indica*	141.76	11	27
	*S. bicolor*	58.56	5	18
	*Z. mays*	51.94	4	19
	**Basal Angiosperms**			
	*A. trichopoda*	266.14	1	1811
	**Gymnosperms**			
	*C. taitungensis*	62.65	2	5070
**Bryophytes**	*M. polymorpha*	2.08	0	13
	*P. patens*	0	0	0

## Supplementary Materials

Supplementary materials can be found at http://www.mdpi.com/1422-0067/19/2/547/s1.

## References

[1] Gray M.W. (2015). Mosaic nature of the mitochondrial proteome: Implications for the origin and evolution of mitochondria. Proc. Natl. Acad. Sci. USA.

[2] Bock R. (2017). Witnessing genome evolution: Experimental reconstruction of endosymbiotic and horizontal gene transfer. Annu. Rev. Genet..

[3] Stern D.B., Lonsdale D.M. (1982). Mitochondrial and chloroplast genomes of maize have a 12-kilobase DNA-sequence in common. Nature.

[4] Knoop V., Unseld M., Marienfeld J., Brandt P., Sunkel S., Ullrich H., Brennicke A. (1996). Copia-, gypsy- and line-like retrotransposon fragments in the mitochondrial genome of *Arabidopsis thaliana*. Genetics.

[5] Wang D., Rousseau-Gueutin M., Timmis J.N. (2012). Plastid sequences contribute to some plant mitochondrial genes. Mol. Biol. Evol..

[6] Liu G.Z., Cao D.D., Li S.S., Su A.G., Geng J.N., Grover C.E., Hu S.N., Hua J.P. (2013). The complete mitochondrial genome of *Gossypium hirsutum* and evolutionary analysis of higher plant mitochondrial genomes. PLoS ONE.

[7] Tang M.Y., Chen Z.W., Grover C.E., Wang Y.M., Li S.S., Liu G.Z., Ma Z.Y., Wendel J.F., Hua J.P. (2015). Rapid evolutionary divergence of *Gossypium barbadense* and *G. hirsutum* mitochondrial genomes. BMC Genom..

[8] Notsu Y., Masood S., Nishikawa T., Kubo N., Akiduki G., Nakazono M., Hirai A., Kadowaki K. (2002). The complete sequence of the rice (*Oryza sativa* L.) mitochondrial genome: Frequent DNA sequence acquisition and loss during the evolution of flowering plants. Mol. Genet. Genom..

[9] Wang D., Wu Y.W., Shih A.C.C., Wu C.S., Wang Y.N., Chaw S.M. (2007). Transfer of chloroplast genomic DNA to mitochondrial genome occurred at least 300 mya. Mol. Biol. Evol..

[10] Rodriguez-Moreno L., Gonzalez V.M., Benjak A., Marti M.C., Puigdomenech P., Aranda M.A., Garcia-Mas J. (2011). Determination of the melon chloroplast and mitochondrial genome sequences reveals that the largest reported mitochondrial genome in plants contains a significant amount of DNA having a nuclear origin. BMC Genom..

[11] Alverson A.J., Rice D.W., Dickinson S., Barry K., Palmer J.D. (2011). Origins and recombination of the bacterial-sized multichromosomal mitochondrial genome of cucumber. Plant Cell.

[12] Chen Z.W., Nie H.S., Grover C.E., Wang Y.M., Li P., Wang M.Y., Pei H.L., Zhao Y.P., Li S.S., Wendel J.F. (2017). Entire nucleotide sequences of *Gossypium raimondii* and *G. arboreum* mitochondrial genomes revealed a-genome species as cytoplasmic donor of the allotetraploid species. Plant Biol..

[13] Dietrich A., Small I., Cosset A., Weil J.H., Marechal-Drouard L. (1996). Editing and import: Strategies for providing plant mitochondria with a complete set of functional transfer rnas. Biochimie.

[14] Clifton S.W., Minx P., Fauron C.M.R., Gibson M., Allen J.O., Sun H., Thompson M., Barbazuk W.B., Kanuganti S., Tayloe C. (2004). Sequence and comparative analysis of the maize NB mitochondrial genome. Plant Physiol..

[15] Sloan D.B., Alverson A.J., Storchova H., Palmer J.D., Taylor D.R. (2010). Extensive loss of translational genes in the structurally dynamic mitochondrial genome of the angiosperm *Silene latifolia*. BMC Evol. Biol..

[16] Iorizzo M., Senalik D., Szklarczyk M., Grzebelus D., Spooner D., Simon P. (2012). De novo assembly of the carrot mitochondrial genome using next generation sequencing of whole genomic DNA provides first evidence of DNA transfer into an angiosperm plastid genome. BMC Plant Biol..

[17] Straub S.C.K., Cronn R.C., Edwards C., Fishbein M., Liston A. (2013). Horizontal transfer of DNA from the mitochondrial to the plastid genome and its subsequent evolution in milkweeds (*Apocynaceae*). Genome Biol. Evol..

[18] Iorizzo M., Grzebelus D., Senalik D., Szklarczyk M., Spooner D., Simon P. (2012). Against the traffic: The first evidence for mitochondrial DNA transfer into the plastid genome. Mob. Genet. Elem..

[19] Liu P.F., Erez A., Nagamani S.C.S., Dhar S.U., Kolodziejska K.E., Dharmadhikari A.V., Cooper M.L., Wiszniewska J., Zhang F., Withers M.A. (2011). Chromosome catastrophes involve replication mechanisms generating complex genomic rearrangements. Cell.

[20] Hastings P.J., Lupski J.R., Rosenberg S.M., Ira G. (2009). Mechanisms of change in gene copy number. Nat. Rev. Genet..

[21] Martin W., Stoebe B., Goremykin V., Hansmann S., Hasegawa M., Kowallik K.V. (1998). Gene transfer to the nucleus and the evolution of chloroplasts. Nature.

[22] Blanchard J.L., Schmidt G.W. (1995). Pervasive migration of organellar DNA to the nucleus in plants. J. Mol. Evol..

[23] Bergthorsson U., Adams K.L., Thomason B., Palmer J.D. (2003). Widespread horizontal transfer of mitochondrial genes in flowering plants. Nature.

[24] Ku C., Nelson-Sathi S., Roettger M., Sousa F.L., Lockhart P.J., Bryant D., Hazkani-Covo E., McInerney J.O., Landan G., Martin W.F. (2015). Endosymbiotic origin and differential loss of eukaryotic genes. Nature.

[25] Chen Z.W., Nie H.S., Wang Y.M., Pei H.L., Li S.S., Zhang L.D., Hua J.P. (2017). Rapid evolutionary divergence of diploid and allotetraploid gossypium mitochondrial genomes. BMC Genom..

[26] Bock R., Timmis J.N. (2008). Reconstructing evolution: Gene transfer from plastids to the nucleus. BioEssays.

[27] Bonnefoy N., Remacle C., Fox T.D. (2007). Genetic transformation of *saccharomyces cerevisiae* and *chlamydomonas reinhardtii* mitochondria. Methods Cell Biol..

[28] Covello P., Gray M.W. (1992). Silent mitochondrial and active nuclear genes for subunit 2 of cytochrome c oxidase (cox2) in soybean: Evidence for rna-mediated gene transfer. EMBO J..

[29] Nugent J.M., Palmer J.D. (1991). RNA-mediated transfer of the gene coxII from the mitochondrion to the nucleus during flowering plant evolution. Cell.

[30] Wang D., Timmis J.N. (2013). Cytoplasmic organelle DNA preferentially inserts into open chromatin. Genome Biol. Evol..

[31] Kudla J., Albertazzi F., Blazević D., Hermann M., Bock R. (2002). Loss of the mitochondrial cox2 intron 1 in a family of monocotyledonous plants and utilization of mitochondrial intron sequences for the construction of a nuclear intron. Mol. Genet. Genom..

[32] Kadowaki K.-I., Kubo N., Ozawa K., Hirai A. (1997). Targeting presequence acquisition after mitochondrial gene transfer to the nucleus occurs by duplication of existing target signals. EMBO J..

[33] Adams K.L., Qiu Y.L., Stoutemyer M., Palmer J.D. (2002). Punctuated evolution of mitochondrial gene content: High and variable rates of mitochondrial gene loss and transfer to the nucleus during angiosperm evolution. Proc. Natl. Acad. Sci. USA.

[34] Adams K.L., Palmer J.D. (2003). Evolution of mitochondrial gene content: Gene loss and transfer to the nucleus. Mol. Phylogenet. Evol..

[35] Wang D., Qu Z.P., Adelson D.L., Zhu J.K., Timmis J.N. (2014). Transcription of nuclear organellar DNA in a model plant system. Genome Biol. Evol..

[36] Goremykin V.V., Salamini F., Velasco R., Viola R. (2009). Mitochondrial DNA of *Vitis vinifera* and the issue of rampant horizontal gene transfer. Mol. Biol. Evol..

[37] Smith D.R. (2014). Mitochondrion-to-plastid DNA transfer: It happens. New Phytol..

[38] Downie S., Jansen R. (2015). A comparative analysis of whole plastid genomes from the *apiales*: Expansion and contraction of the inverted repeat, mitochondrial to plastid transfer of DNA and identification of highly divergent noncoding regions. Syst. Bot..

[39] Spooner D.M., Ruess H., Iorizzo M., Senalik D., Simon P. (2017). Entire plastid phylogeny of the carrot genus (*Daucus*, *Apiaceae*): Concordance with nuclear data and mitochondrial and nuclear DNA insertions to the plastid. Am. J. Bot..

[40] Ma P.F., Zhang Y.X., Guo Z.H., Li D.Z. (2015). Evidence for horizontal transfer of mitochondrial DNA to the plastid genome in a bamboo genus. Sci. Rep..

[41] Wysocki W.P., Clark L.G., Attigala L., Ruiz-Sanchez E., Duvall M.R. (2015). Evolution of the bamboos (*Bambusoideae*; *Poaceae*): A full plastome phylogenomic analysis. BMC Evol. Biol..

[42] Smith D.R. (2011). Extending the limited transfer window hypothesis to inter-organelle DNA migration. Genome Biol. Evol..

[43] Cerutti H., Jagendorf A. (1995). Movement of DNA across the chloroplast envelope: Implications for the transfer of promiscuous DNA. Photosynth. Res..

[44] Wang D., Lloyd A.H., Timmis J.N. (2012). Environmental stress increases the entry of cytoplasmic organellar DNA into the nucleus in plants. Proc. Natl. Acad. Sci. USA.

[45] Kurland C.G., Andersson S.G.E. (2000). Origin and evolution of the mitochondrial proteome. Microbiol. Mol. Biol. Rev..

[46] Kitazaki K., Kubo T. (2010). Cost of having the largest mitochondrial genome: Evolutionary mechanism of plant mitochondrial genome. J. Bot..

[47] Ong H.C., Palmer J.D. (2006). Pervasive survival of expressed mitochondrial *rps14* pseudogenes in grasses and their relatives for 80 million years following three functional transfers to the nucleus. BMC Evol. Biol..

[48] Pamilo P., Viljakainen L., Vihavainen A. (2007). Exceptionally high density of numts in the honeybee genome. Mol. Biol. Evol..

[49] Timmis J.N., Ayliffe M.A., Huang C.Y., Martin W. (2004). Endosymbiotic gene transfer: Organelle genomes forge eukaryotic chromosomes. Nat. Rev. Genet..

[50] Wang X., Wang H., Wang J., Sun R., Wu J., Liu S., Bai Y., Mun J.H., Bancroft I., Cheng F. (2011). The genome of the mesopolyploid crop species *Brassica rapa*. Nat. Genet..

[51] Yang J., Liu D., Wang X., Ji C., Cheng F. (2016). The genome sequence of allopolyploid *Brassica juncea* and analysis of differential homoeolog gene expression influencing selection. Nat. Genet..

[52] Liu S., Liu Y., Yang X., Tong C., Edwards D., Parkin I.A., Zhao M., Ma J., Yu J., Huang S. (2014). The *Brassica oleracea* genome reveals the asymmetrical evolution of polyploid genomes. Nat. Commun..

[53] Pucker B., Holtgrawe D., Rosleff Sorensen T., Stracke R., Viehover P., Weisshaar B. (2016). A de novo genome sequence assembly of the *Arabidopsis thaliana* accession niederzenz-1 displays presence/absence variation and strong synteny. PLoS ONE.

[54] Ming R., Hou S., Feng Y., Yu Q., Dionne-Laporte A., Saw J.H., Senin P., Wang W., Ly B.V., Lewis K.L. (2008). The draft genome of the transgenic tropical fruit tree papaya (*Carica papaya* Linnaeus). Nature.

[55] Chan A.P., Crabtree J., Zhao Q., Lorenzi H., Orvis J., Puiu D., Melake-Berhan A., Jones K.M., Redman J., Chen G. (2010). Draft genome sequence of the oilseed species *Ricinus communis*. Nat. Biotechnol..

[56] Guo S., Zhang J., Sun H., Salse J., Lucas W.J., Zhang H., Zheng Y., Mao L., Ren Y., Wang Z. (2013). The draft genome of watermelon (*Citrullus lanatus*) and resequencing of 20 diverse accessions. Nat. Genet..

[57] Schmutz J., Cannon S.B., Schlueter J., Ma J., Mitros T., Nelson W., Hyten D.L., Song Q., Thelen J.J., Cheng J. (2010). Genome sequence of the palaeopolyploid soybean. Nature.

[58] Kang Y.J., Kim S.K., Kim M.Y., Lestari P., Kim K.H., Ha B.K., Jun T.H., Hwang W.J., Lee T., Lee J. (2014). Genome sequence of mungbean and insights into evolution within vigna species. Nat. Commun..

[59] Cegan R., Vyskot B., Kejnovsky E., Kubat Z., Blavet H., Safar J., Dolezel J., Blavet N., Hobza R. (2012). Genomic diversity in two related plant species with and without sex chromosomes—*Silene latifolia* and *S. vulgaris*. PLoS ONE.

[60] Iorizzo M., Ellison S., Senalik D. (2016). A high-quality carrot genome assembly provides new insights into carotenoid accumulation and asterid genome evolution. Nat. Genet..

[61] Sierro N., Battey J.N., Ouadi S., Bakaher N., Bovet L., Willig A., Goepfert S., Peitsch M.C., Ivanov N.V. (2014). The tobacco genome sequence and its comparison with those of tomato and potato. Nat. Commun..

[62] Jaillon O., Aury J.M., Noel B., Policriti A., Clepet C., Casagrande A., Choisne N., Aubourg S., Vitulo N., Jubin C. (2007). The grapevine genome sequence suggests ancestral hexaploidization in major angiosperm phyla. Nature.

[63] Wang W., Haberer G., Gundlach H., Glasser C., Nussbaumer T., Luo M.C., Lomsadze A., Borodovsky M., Kerstetter R.A., Shanklin J. (2014). The *Spirodela polyrhiza* genome reveals insights into its neotenous reduction fast growth and aquatic lifestyle. Nat. Commun..

[64] Al-Mssallem I.S., Hu S., Zhang X., Lin Q., Liu W., Tan J., Yu X., Liu J., Pan L., Zhang T. (2013). Genome sequence of the date palm *Phoenix dactylifera* L. Nat. Commun..

[65] Goff S.A., Ricke D., Lan T.H., Presting G., Wang R., Dunn M., Glazebrook J., Sessions A., Oeller P., Varma H. (2002). A draft sequence of the rice genome (*Oryza sativa* L. ssp. *japonica*). Science.

[66] Zhang J., Chen L.L., Xing F., Kudrna D.A., Yao W., Copetti D., Mu T., Li W., Song J.M., Xie W. (2016). Extensive sequence divergence between the reference genomes of two elite indica rice varieties zhenshan 97 and minghui 63. Proc. Natl. Acad. Sci. USA.

[67] Paterson A.H., Bowers J.E., Bruggmann R., Dubchak I., Grimwood J., Gundlach H., Haberer G., Hellsten U., Mitros T., Poliakov A. (2009). The *Sorghum bicolor* genome and the diversification of grasses. Nature.

[68] Schnable P.S., Ware D., Fulton R.S., Stein J.C., Wei F., Pasternak S., Liang C., Zhang J., Fulton L., Graves T.A. (2009). The B73 maize genome: Complexity, diversity and dynamics. Science.

[69] Project A.G. (2013). The *Amborella* genome and the evolution of flowering plants. Science.

[70] Izuno A., Hatakeyama M., Nishiyama T., Tamaki I., Shimizu-Inatsugi R., Sasaki R., Shimizu K.K., Isagi Y. (2016). Genome sequencing of *Metrosideros polymorpha* (Myrtaceae), a dominant species in various habitats in the hawaiian islands with remarkable phenotypic variations. J. Plant Res..

[71] Rensing S.A., Lang D., Zimmer A.D., Terry A., Salamov A., Shapiro H., Nishiyama T., Perroud P.F., Lindquist E.A., Kamisugi Y. (2008). The *Physcomitrella* genome reveals evolutionary insights into the conquest of land by plants. Science.

[72] Qiu F., Ungerer M.C. (2018). Genomic abundance and transcriptional activity of diverse gypsy and copia long terminal repeat retrotransposons in three wild sunflower species. BMC Plant Biol..

[73] Goremykin V.V., Lockhart P.J., Viola R., Velasco R. (2012). The mitochondrial genome of *Malus domestica* and the import-driven hypothesis of mitochondrial genome expansion in seed plants. Plant J..

[74] Zhang J., Ruhlman T.A., Sabir J., Blazier J.C., Jansen R.K. (2015). Coordinated rates of evolution between interacting plastid and nuclear genes in *Geraniaceae*. Plant Cell.

[75] Sugiura C., Kobayashi Y., Aoki S., Sugita C., Sugita M. (2003). Complete chloroplast DNA sequence of the moss *Physcomitrella patens*: Evidence for the loss and relocation of *rpoa* from the chloroplast to the nucleus. Nucleic Acids Res..

[76] Altschul S.F., Gish W., Miller W., Myers E.W., Lipman D.J. (1990). Basic local alignment search tool. J. Mol. Biol..

[77] Hazkani-Covo E., Covo S. (2008). Numt-mediated double-strand break repair mitigates deletions during primate genome evolution. PLoS Genet..

[78] Ju Y.S., Tubio J.M.C., Mifsud W., Fu B.Y., Davies H.R., Ramakrishna M., Li Y.L., Yates L., Gundem G., Tarpey P.S. (2015). Frequent somatic transfer of mitochondrial DNA into the nuclear genome of human cancer cells. Genome Res..

[79] Tamura K., Peterson D., Peterson N., Stecher G., Nei M., Kumar S. (2011). Mega5: Molecular evolutionary genetics analysis using maximum likelihood, evolutionary distance and maximum parsimony methods. Mol. Biol. Evol..

[80] Chen Z.W., Feng K., Grover C.E., Li P.B., Liu F., Wang Y.M., Xu Q., Shang M.Z., Zhou Z.L., Cai X.Y. (2016). Chloroplast DNA structural variation, phylogeny and age of divergence among diploid cotton species. PLoS ONE.

[81] Chen Z.W., Grover C.E., Li P.B., Wang Y.M., Nie H.S., Zhao Y.P., Wang M.Y., Liu F., Zhou Z.L., Wang X.X. (2017). Molecular evolution of the plastid genome during diversification of the cotton genus. Mol. Phylogenet. Evol..

