# Supplementary material for: The Roles of Mitochondrion in Intergenomic Gene Transfer in Plants: A Source and a Pool
Source: Int J Mol Sci. 2018 Feb 11;19(2):547. doi: 10.3390/ijms19020547 (PMC5855769; doi:10.3390/ijms19020547)
Supplement: Supplementary file 1 [file ijms-19-00547-s001.zip › ijms-260413 - Supplementary Figures and Tables/Figure S1.docx]

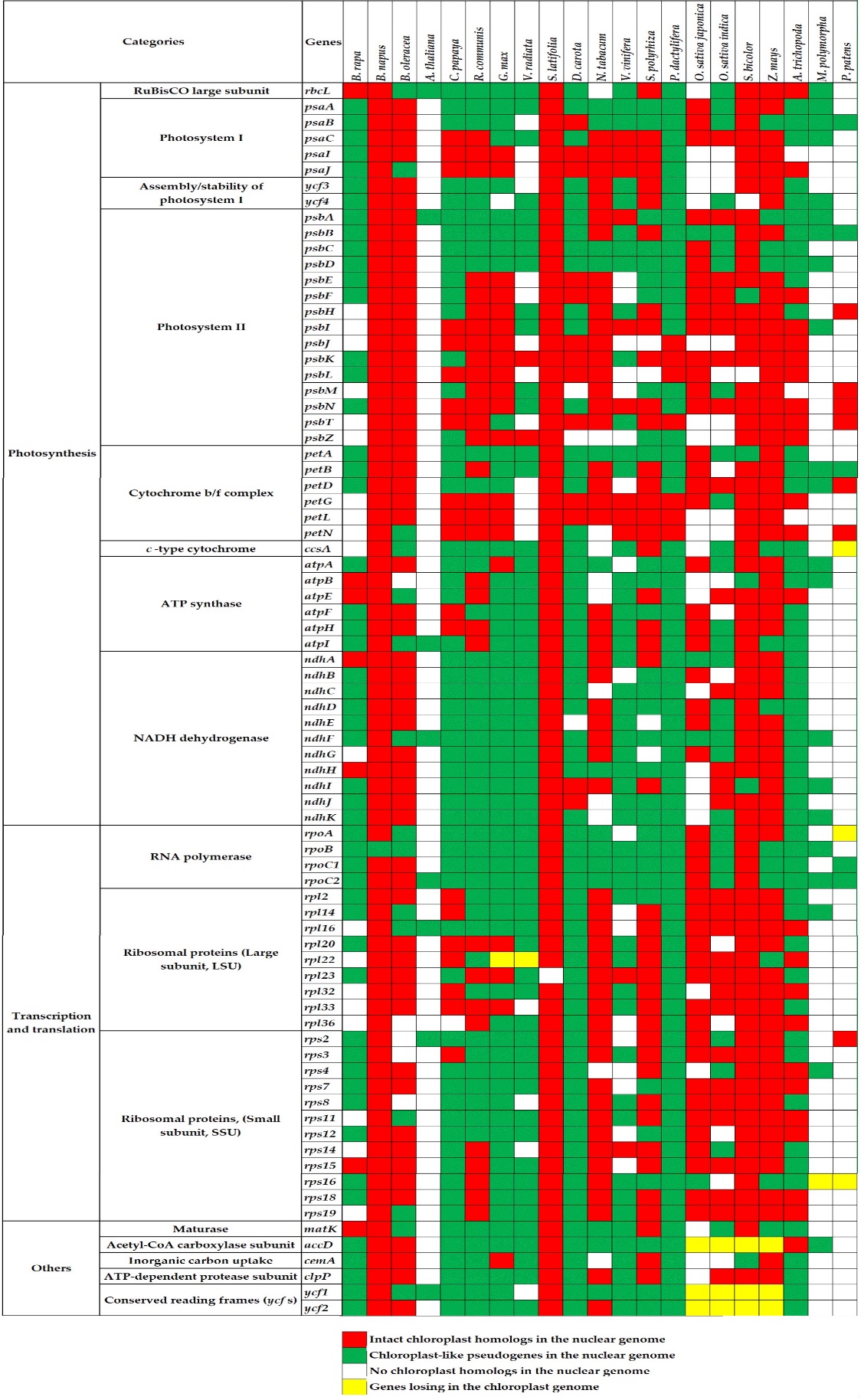


**Figure S1.** Genes identified to transfer in and out of the chloroplast genome or genes lost from the chloroplast genome of 21 land plants. The first three columns are mitochondrial protein-encoding genes (the third column) and their functional categories (the first two columns). The first line lists the names of plant species. The red and green cells represent chloroplast full-length intact homologs and pseudogenes in nuclear genomes, respectively. The white and yellow cells represent no chloroplast homologs in nuclear genomes and genes lost from chloroplast genomes, respectively.
