# Supplementary material for: The Roles of Mitochondrion in Intergenomic Gene Transfer in Plants: A Source and a Pool
Source: Int J Mol Sci. 2018 Feb 11;19(2):547. doi: 10.3390/ijms19020547 (PMC5855769; doi:10.3390/ijms19020547)
Supplement: Supplementary file 1 [file ijms-19-00547-s001.zip › ijms-260413 - Supplementary Figures and Tables/Figure S1.pdf]

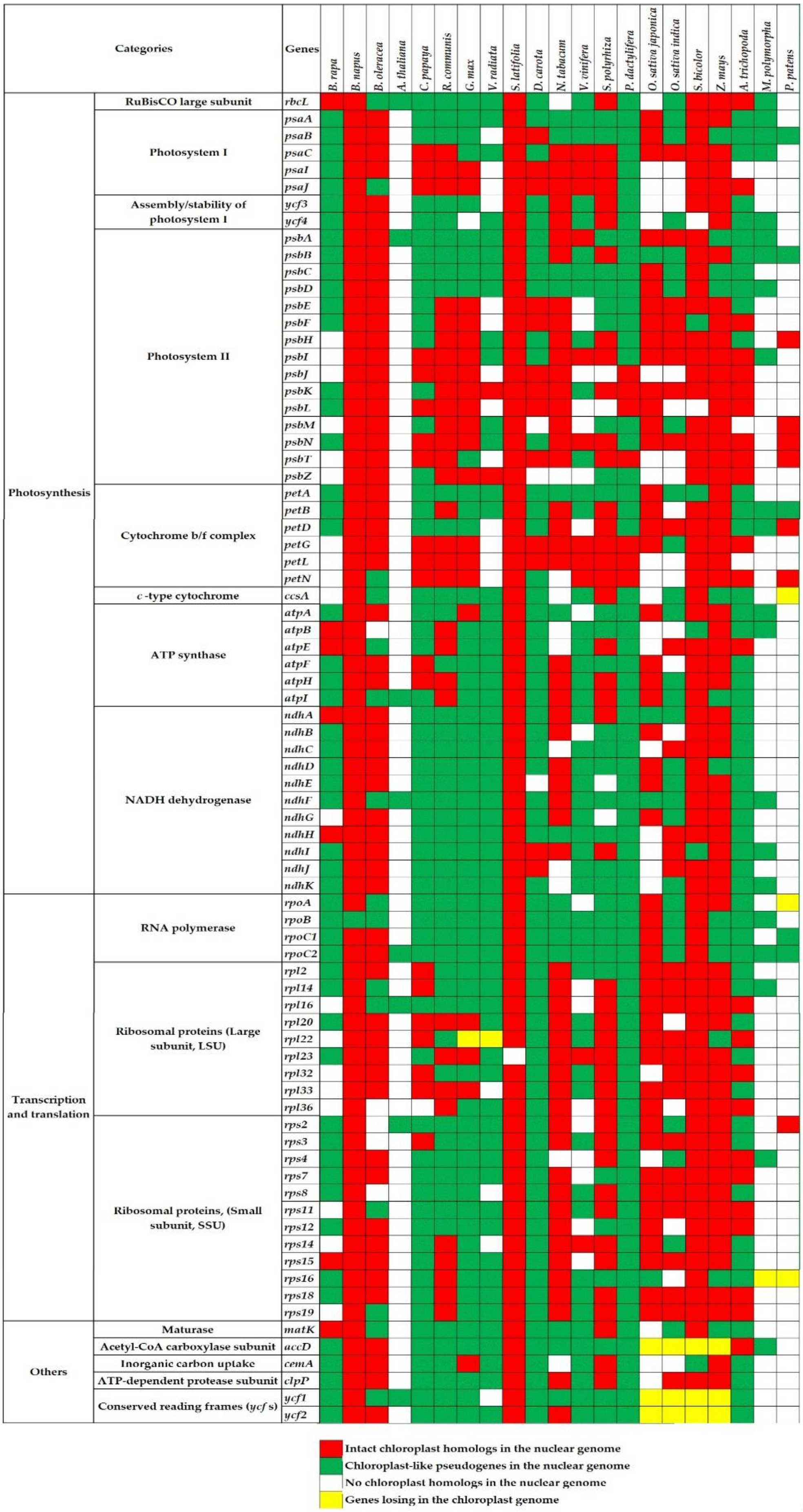

Categories
Genes
B. rapa
B. napus
B. oleracea
A. thaliana
C. papaya
R. communis
G. max
V. radiata
S. latifolia
D. carota
N. tabacum
V. vinifera
S. polyrhiza
P. dactylifera
O. sativa japonica
O. sativa indica
S. bicolor
Z. mays
A. trichopoda
M. polymorpha
P. patens
Photosynthesis
RuBisCO large subunit
rbcL
Photosystem I
psaA
psaB
psaC
psaI
psaJ
Assembly/stability of photosystem I
ycf3
ycf4
Photosystem II
psbA
psbB
psbC
psbD
psbE
psbF
psbH
psbI
psbJ
psbK
psbL
psbM
psbN
psbT
psbZ
Cytochrome b/f complex
petA
petB
petD
petG
petL
petN
c-type cytochrome
ccsA
ATP synthase
atpA
atpB
atpE
atpF
atpH
atpI
NADH dehydrogenase
ndhA
ndhB
ndhC
ndhD
ndhE
ndhF
ndhG
ndhH
ndhI
ndhJ
ndhK
Transcription and translation
RNA polymerase
rpoA
rpoB
rpoC1
rpoC2
Ribosomal proteins (Large subunit, LSU)
rpl2
rpl14
rpl16
rpl20
rpl22
rpl23
rpl32
rpl33
rpl36
Ribosomal proteins, (Small subunit, SSU)
rps2
rps3
rps4
rps7
rps8
rps11
rps12
rps14
rps15
rps16
rps18
rps19
Others
Maturase
matK
Acetyl-CoA carboxylase subunit
accD
Inorganic carbon uptake
cemA
ATP-dependent protease subunit
clpP
Conserved reading frames (ycf s)
ycf1
ycf2
Intact chloroplast homologs in the nuclear genome
Chloroplast-like pseudogenes in the nuclear genome
No chloroplast homologs in the nuclear genome
Genes losing in the chloroplast genome

Figure S1. Genes identified to transfer in and out of the chloroplast genome or genes lost from the chloroplast genome of 21 land plants. The first three columns are mitochondrial protein-encoding genes (the third column) and their functional categories (the first two columns). The first line lists the names of plant species. The red and green cells represent chloroplast full-length intact homologs and pseudogenes in nuclear genomes, respectively. The white and yellow cells represent no chloroplast homologs in nuclear genomes and genes lost from chloroplast genomes, respectively.
