# Supplementary material for: The Roles of Mitochondrion in Intergenomic Gene Transfer in Plants: A Source and a Pool
Source: Int J Mol Sci. 2018 Feb 11;19(2):547. doi: 10.3390/ijms19020547 (PMC5855769; doi:10.3390/ijms19020547)
Supplement: Supplementary file 1 [file ijms-19-00547-s001.zip › ijms-260413 - Supplementary Figures and Tables/Figure S2.pdf]

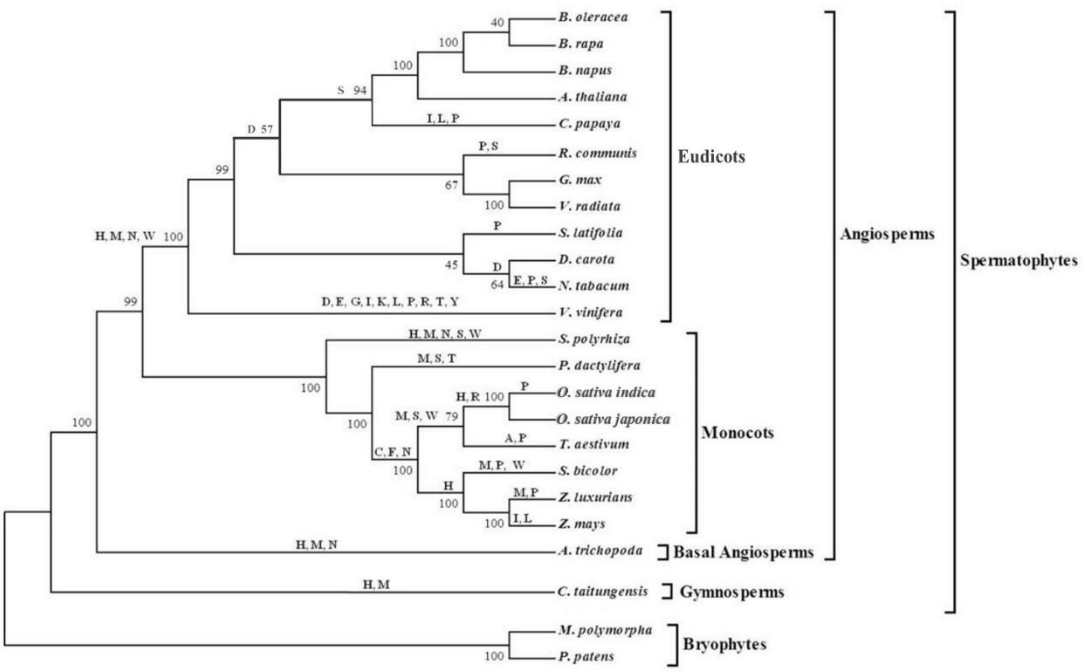

Figure S2. Phylogenetic analysis of chloroplast-derived tRNA genes losing and transferring to the mitochondrion in land plants. The capital letters at the nodes and the branches denote the corresponding amino acids encoded by chloroplast-like tRNA genes in mitochondrial genomes. A: Alanine; C: Cysteine; D: Aspartic acid; E: Glutamic acid; F: Phenylalanine; G: Glicine; H: Histidine; I: Isoleucine; K: Lysine; L: Leucine; M: Methionine; N: Asparagine; P: Proline; Q: Glutanine; R: Arginine; S: Serine; T: Threonine; V: Valine; W: Tryptophan; Y: Tyrosine. The number at the nodes represent bootstrap values. The phylogenetic tree is constructed by the maximum likelihood (ML) method with the model GTR + G + I.
