# Supplementary material for: The Roles of Mitochondrion in Intergenomic Gene Transfer in Plants: A Source and a Pool
Source: Int J Mol Sci. 2018 Feb 11;19(2):547. doi: 10.3390/ijms19020547 (PMC5855769; doi:10.3390/ijms19020547)
Supplement: Supplementary file 1 [file ijms-19-00547-s001.zip › ijms-260413 - Supplementary Figures and Tables/Figure S4.pdf]

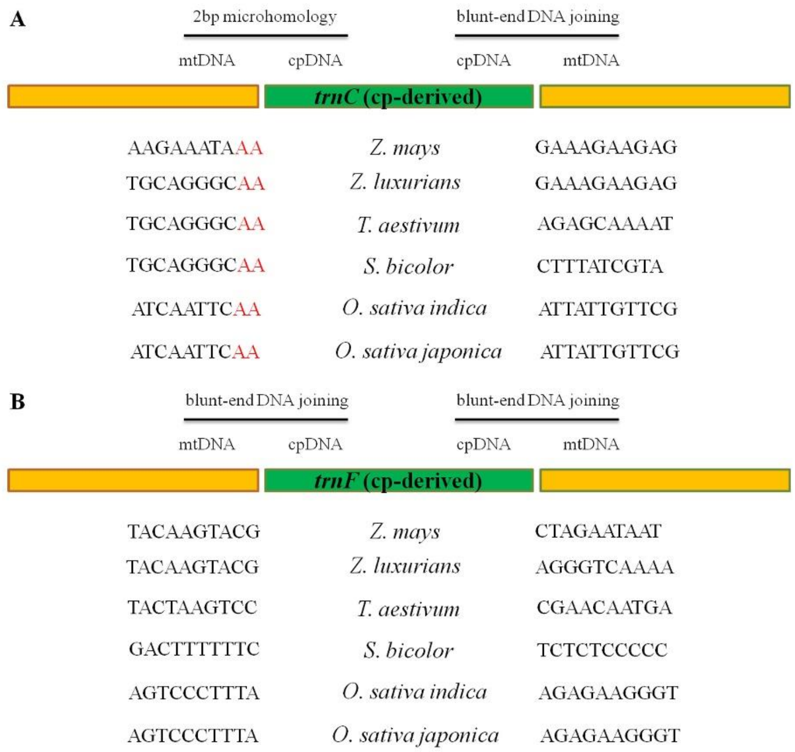

Figure S4. Nucleotide-resolution analysis on flanking sequences of chloroplast-derived *trnC* and *trnF* genes in mitochondrial genomes of most *Gramineae* crops. Red color indicates nucleotide sequence micro-homologies. cpDNA and mtDNA are the abbreviations of chloroplast DNA and mitochondrial DNA, respectively. The yellow-green-yellow strip represents the fusion sequence of mtDNA-cpDNA-mtDNA. The sequences under the two yellow strips on the left and right are the flanking sequences of inserted chloroplast-like tRNA gene in the mitochondrial genomes. The red capital English letters close to cpDNA indicate the nucleotides of micro-homologies among the different species.
