# Supplementary material for: The Roles of Mitochondrion in Intergenomic Gene Transfer in Plants: A Source and a Pool
Source: Int J Mol Sci. 2018 Feb 11;19(2):547. doi: 10.3390/ijms19020547 (PMC5855769; doi:10.3390/ijms19020547)
Supplement: Supplementary file 1 [file ijms-19-00547-s001.zip › ijms-260413 - Supplementary Figures and Tables/Table S1.docx]

**Table S1.** Accession numbers for nuclear, mitochondrial and chloroplast genomes of 24 plants species used in present research.

| **Species** | | **Nuclear genome Accession No.** | | **Mitochondrial genome Accession No.** | | | | | **Chloroplast genome Accession No.** |
| --- | --- | --- | --- | --- | --- | --- | --- | --- | --- |
| **Spermatophytes** | **Eudicots** | | | | | | | | |
|  | *Brassica rapa* (*B. rapa*) | | AENI00000000 | | JF920285 | | | DQ231548 | |
|  | *Brassica napus* (*B. napus*) | | PRJNA293435 | | AP006444 | | | GQ861354 | |
|  | *Brassica oleracea* (*B. oleracea*) | | AOIX00000000 | | JF920286 | | | n.a.^1^ | |
|  | *Arabidopsis thaliana* (*A. thaliana*) | | PRJNA10719 | | Y08501 | | | AP000423 | |
|  | *Carica papaya* (*C. papaya*) | | ABIM00000000 | | EU431224 | | | EU431223 | |
|  | *Ricinus communis* (*R. communis*) | | AASG02000000 | | HQ874649 | | | JF937588 | |
|  | *Glycine max* (*G. max*) | | ACUP00000000 | | JX463295 | | | DQ317523 | |
|  | *Vigna radiate* (*V. radiata*) | | PRJNA301363 | | HM367685 | | | GQ893027 | |
|  | *Silene latifolia* (*S. latifolia*) | | PRJNA289891 | | HM562727 | | | JF715055 | |
|  | *Daucus carota* (*D. carota*) | | PRJNA326436 | | JQ248574 | | | DQ898156 | |
|  | *Nicotiana tabacum* (*N. tabacum*) | | PRJNA170566 | | BA000042 | | | Z00044 | |
|  | *Vitis vinifera* (*V. vinifera*) | | PRJEA18785 | | FM179380 | | | DQ424856 | |
|  | **Monocots** | | | | | | | | |
|  | *Spirodela polyrhiza* (*S. polyrhiza*) | | PRJNA205940 | | JQ804980 | | | JN160603 | |
|  | *Phoenix dactylifera* (*P. dactylifera*) | | PRJNA249070 | | JN375330 | | | GU811709 | |
|  | *Oryza sativa* *Japonica* (*O. sativa japonica*) | | PRJNA12269 | | BA000029 | | | X15901 | |
|  | *Oryza sativa indica* (*O. sativa indica*) | | PRJNA361 | | DQ167399 | | | AY522329 | |
|  | *Triticum aestivum* (*T. aestivum*) | | PRJEB11773 | | AP008982 | | | AB042240 | |
|  | *Sorghum bicolor* (*S. bicolor*) | | PRJNA13876 | | DQ984518 | | | EF115542 | |
|  | *Zea luxurians* (*Z. luxurians*) | | n.a.^1^ | | DQ645537 | | | KR873424 | |
|  | *Zea mays* (*Z. mays*) | | PRJNA10769 | | AY506529 | | | X86563 | |
|  | **Basal Angiosperms** | | | | | | | | |
|  | *Amborella trichopoda* (*A. trichopoda*) | | PRJNA212863 | | KF754799-KF754803 | | | AJ506156 | |
|  | **Gymnosperms** | | | | | | | | |
|  | *Cycas taitungensis* (*C. taitungensis*) | | n.a.^1^ | | AP009381 | | | AP009339 | |
| **Bryophytes** | | | | | | | | | |
|  | *Marchantia polymorpha* (*M. polymorpha*) | | PRJNA310693 | | | M68929 | | X04465 | |
|  | *Physcomitrella patens* (*P. patens*) | ABEU01000000 | | | | | AB251495 | AP005672 | |

^1^ “n. a.” notes the genome not released yet.
