# Supplementary material for: The Roles of Mitochondrion in Intergenomic Gene Transfer in Plants: A Source and a Pool
Source: Int J Mol Sci. 2018 Feb 11;19(2):547. doi: 10.3390/ijms19020547 (PMC5855769; doi:10.3390/ijms19020547)
Supplement: Supplementary file 1 [file ijms-19-00547-s001.zip › ijms-260413 - Supplementary Figures and Tables/Table S1.pdf]

Table S1. Accession numbers for nuclear, mitochondrial and chloroplast genomes of 24 plants species used in present research.

Species		Nuclear genome Accession No.	Mitochondrial genome Accession No.	Chloroplast genome Accession No.
**Spermatophytes**	**Eudicots**			
	Brassica rapa (*B. rapa*)	AENI00000000	JF920285	DQ231548
	Brassica napus (*B. napus*)	PRJNA293435	AP006444	GQ861354
	Brassica oleracea (*B. oleracea*)	AOIX00000000	JF920286	n.a.[1]
	Arabidopsis thaliana (*A. thaliana*)	PRJNA10719	Y08501	AP000423
	Carica papaya (*C. papaya*)	ABIM00000000	EU431224	EU431223
	Ricinus communis (*R. communis*)	AASG02000000	HQ874649	JF937588
	Glycine max (*G. max*)	ACUP00000000	JX463295	DQ317523
	Vigna radiate (*V. radiata*)	PRJNA301363	HM367685	GQ893027
	Silene latifolia (*S. latifolia*)	PRJNA289891	HM562727	JF715055
	Daucus carota (*D. carota*)	PRJNA326436	JQ248574	DQ898156
	Nicotiana tabacum (*N. tabacum*)	PRJNA170566	BA000042	Z00044
	Vitis vinifera (*V. vinifera*)	PRJEA18785	FM179380	DQ424856
	**Monocots**			
	Spirodela polyrhiza (*S. polyrhiza*)	PRJNA205940	JQ804980	JN160603
	Phoenix dactylifera (*P. dactylifera*)	PRJNA249070	JN375330	GU811709
	Oryza sativa Japonica (*O. sativa japonica*)	PRJNA12269	BA000029	X15901
	Oryza sativa indica (*O. sativa indica*)	PRJNA361	DQ167399	AY522329
	Triticum aestivum (*T. aestivum*)	PRJEB11773	AP008982	AB042240
	Sorghum bicolor (*S. bicolor*)	PRJNA13876	DQ984518	EF115542
	Zea luxurians (*Z. luxurians*)	n.a.[1]	DQ645537	KR873424
	Zea mays (*Z. mays*)	PRJNA10769	AY506529	X86563
	**Basal Angiosperms**			
	Amborella trichopoda (*A. trichopoda*)	PRJNA212863	KF754799-KF754803	AJ506156
	**Gymnosperms**			
	Cycas taitungensis (*C. taitungensis*)	n.a.[1]	AP009381	AP009339
**Bryophytes**				
	Marchantia polymorpha (*M. polymorpha*)	PRJNA310693	M68929	X04465
	Physcomitrella patens (*P. patens*)	ABEU01000000	AB251495	AP005672

[1] "n. a." notes the genome not released yet.
