# Supplementary material for: The Roles of Mitochondrion in Intergenomic Gene Transfer in Plants: A Source and a Pool
Source: Int J Mol Sci. 2018 Feb 11;19(2):547. doi: 10.3390/ijms19020547 (PMC5855769; doi:10.3390/ijms19020547)
Supplement: Supplementary file 1 [file ijms-19-00547-s001.zip › ijms-260413 - Supplementary Figures and Tables/Table S2.docx]

**Table S2.** Total length (bp) of nuclear-transferred sequences in mitochondrial genomes of 23 plants.

| **Species** | | **Copia** | | **Gypsy** | **Low**  **complexity** | **LTR-**  **retro** | | | | **Simple**  **repeat** | **TE** | **Un-**  **specified** | | | | | **Total length** | |
| --- | --- | --- | --- | --- | --- | --- | --- | --- | --- | --- | --- | --- | --- | --- | --- | --- | --- | --- |
| **Spermatophytes** | **Eudicots** |  |  |  |  |  |  |  |  |  |  |  |  |  |  |  |  |  |
|  | *B. rapa* | 3,157 | | 2,288 | 136 | | **5,039^1^** | | 550 | | 1,650 | | 428 | | | | 13,248 | |
|  | *B. napus* | 428 | | 2,288 | 30 | | **5,137^1^** | | 657 | | 1,588 | | 428 | | | | 10,556 | |
|  | *B. oleracea* | 6,416 | | 3,583 | 342 | | 8,799**^1^** | | 731 | | 3,004 | | 797 | | | | 23,672 | |
|  | *A. thaliana* | **8,422^1^** | | 6,206 | 217 | | 3,606 | | 1,140 | | 1,792 | | 369 | | | | 21,752 | |
|  | *C. papaya* | 2,804 | | 3,586 | 350 | | 15,279**^1^** | | 1,490 | | 2,008 | | 309 | | | | 25,826 | |
|  | *R. communis* | 1,416 | | **7,543^1^** | 333 | | 2,943 | | 1,814 | | 2,104 | | 616 | | | | 16,769 | |
|  | *G. max* | 1,300 | | 3,798 | 195 | | **5,926^1^** | | 849 | | 1,889 | | 620 | | | | 14,577 | |
|  | *V. radiata* | 1,063 | | **4,181^1^** | 3,195 | | 456 | | 738 | | 1,927 | | 649 | | | | 12,209 | |
|  | *S. latifolia* | **2,787^1^** | | n.a.^2^ | 418 | | 1,280 | | 2,110 | | 996 | | 369 | | | | 7,960 | |
|  | *D. carota* | 844 | | **6,431^1^** | 168 | | 4,640 | | 690 | | 2,375 | | 1,006 | | | | 16,154 | |
|  | *N. tabacum* | 3,227 | | 2,953 | 402 | | **9,730^1^** | | 999 | | 4,421 | | 1,404 | | | | 23,136 | |
|  | *V. vinifera* | 2,047 | | **13,502^1^** | 1,024 | | 8,953 | | 4,102 | | 5,802 | | 693 | | | | 36,123 | |
|  | **Monocots** |  |  |  |  |  |  |  |  |  |  |  |  |  |  |  |  |  |
|  | *S. polyrhiza* | 480 | | **4,697^1^** | 113 | | 3,274 | | 869 | | 1,354 | | 504 | | | | 11,291 | |
|  | *P. dactylifera* | 4,818 | | 5,517 | 950 | | **10,058^1^** | | 3,737 | | 2,698 | | 575 | | | | 28,353 | |
|  | *O. sativa japonica* | | 3,557 | 5,461 | 554 | | **6,804^1^** | | 1,201 | | 3,118 | | 738 | | | | 21,433 | |
|  | *O. sativa indica* | 4,509 | | 4,921 | 588 | | 6,824**^1^** | | 1,245 | | 3,134 | | | | 738 | 21,959 | | |
|  | *T. aestivum* | 4,180 | | **8,755^1^** | 372 | | 8,460 | | 1,061 | | 2,188 | | | | 738 | 25,754 | |  |
|  | *S. bicolor* | 2,145 | | 2,486 | 324 | | **5,484^1^** | | 1,473 | | 1,874 | | | | 369 | 14,155 | |  |
|  | *Z. luxurians* | **4,053^1^** | | 436 | 504 | | 3,683 | | 2,732 | | 1,797 | | | | 2,639 | 15,844 | |  |
|  | *Z. mays* | 2,035 | | **10,303^1^** | 370 | | 5,613 | | 2,950 | | 1,634 | | | | 952 | 23,857 | |  |
|  | **Gymnosperms** |  |  |  |  |  |  |  |  |  |  |  |  |  |  |  |  |  |
|  | *C. taitungensis* | 2,889 | | 588 | 78 | | **3,800^1^** | | 2,664 | | 1,182 | | | 369 | | 11,570 | |  |
| **Bryophytes** |  |  |  |  |  |  |  |  |  |  |  |  |  |  |  |  |  |  |
|  | *M. polymorpha* | 228 | | 650 | 133 | | **1,381^1^** | 1,082 | | | 971 | | | 369 | | 4,814 | | |
|  | *P. patens* | 208 | | 613 | 186 | | 1,477**^1^** | 456 | | | 817 | | | 492 | | *4,249*^4^ | | |

^1^ Bold notes the maximum of 7 repeats in each plant species. LTR-retro: long terminal repeat retrotransposons; TE: transposable element.
