# Supplementary material for: The Roles of Mitochondrion in Intergenomic Gene Transfer in Plants: A Source and a Pool
Source: Int J Mol Sci. 2018 Feb 11;19(2):547. doi: 10.3390/ijms19020547 (PMC5855769; doi:10.3390/ijms19020547)
Supplement: Supplementary file 1 [file ijms-19-00547-s001.zip › ijms-260413 - Supplementary Figures and Tables/Table S2.pdf]

Table S2. Total length (bp) of nuclear-transferred sequences in mitochondrial genomes of 23 plants.

Species		Copia	Gypsy	Low complexity	LTR-retro	Simple repeat	TE	Un-specified	Total length
**Spermatophytes**	**Eudicots**								
	B. rapa	3,157	2,288	136	**5,039**[1]	550	1,650	428	13,248
	B. napus	428	2,288	30	**5,137**[1]	657	1,588	428	10,556
	B. oleracea	6,416	3,583	342	8,799[1]	731	3,004	797	23,672
	A. thaliana	**8,422**[1]	6,206	217	3,606	1,140	1,792	369	21,752
	C. papaya	2,804	3,586	350	15,279[1]	1,490	2,008	309	25,826
	R. communis	1,416	**7,543**[1]	333	2,943	1,814	2,104	616	16,769
	G. max	1,300	3,798	195	**5,926**[1]	849	1,889	620	14,577
	V. radiata	1,063	**4,181**[1]	3,195	456	738	1,927	649	12,209
	S. latifolia	**2,787**[1]	n.a.[2]	418	1,280	2,110	996	369	7,960
	D. carota	844	**6,431**[1]	168	4,640	690	2,375	1,006	16,154
	N. tabacum	3,227	2,953	402	**9,730**[1]	999	4,421	1,404	23,136
	V. vinifera	2,047	**13,502**[1]	1,024	8,953	4,102	5,802	693	36,123
	**Monocots**								
	S. polyrhiza	480	**4,697**[1]	113	3,274	869	1,354	504	11,291
	P. dactylifera	4,818	5,517	950	**10,058**[1]	3,737	2,698	575	28,353
	O. sativa japonica	3,557	5,461	554	**6,804**[1]	1,201	3,118	738	21,433
	O. sativa indica	4,509	4,921	588	6,824[1]	1,245	3,134	738	21,959
	T. aestivum	4,180	**8,755**[1]	372	8,460	1,061	2,188	738	25,754
	S. bicolor	2,145	2,486	324	**5,484**[1]	1,473	1,874	369	14,155
	Z. luxurians	**4,053**[1]	436	504	3,683	2,732	1,797	2,639	15,844
	Z. mays	2,035	**10,303**[1]	370	5,613	2,950	1,634	952	23,857
	**Gymnosperms**								
	C. taitungensis	2,889	588	78	**3,800**[1]	2,664	1,182	369	11,570
**Bryophytes**									
	M. polymorpha	228	650	133	**1,381**[1]	1,082	971	369	4,814
	P. patens	208	613	186	1,477[1]	456	817	492	*4,249*[4]

[1] Bold notes the maximum of 7 repeats in each plant species. LTR-retro: long terminal repeat retrotransposons; TE: transposable element.
