# Supplementary material for: The Roles of Mitochondrion in Intergenomic Gene Transfer in Plants: A Source and a Pool
Source: Int J Mol Sci. 2018 Feb 11;19(2):547. doi: 10.3390/ijms19020547 (PMC5855769; doi:10.3390/ijms19020547)
Supplement: Supplementary file 1 [file ijms-19-00547-s001.zip › ijms-260413 - Supplementary Figures and Tables/Table S3.docx]

Table S3. The list of the full names of the analyzed genes and appropriate abbreviations.

| **Organelles** | **Category** | **Genes** |
| --- | --- | --- |
| Mitochondrion | **Respiration chain complexes** | |
|  | Complex I (Nicotinamide adenine dinucleotide dehydrogenase, NADH) | *nad1*, *nad2*, *nad3*, *nad4*, *nad4L*, *nad5*, *nad6*, *nad7*, *nad9* |
|  | Complex II (Succinate dehydrogenase, SDH) | *sdh3*, *sdh4* |
|  | Complex III (Cytochrome b) | *cob* |
|  | Complex IV (Cytochrome c oxidase) | *cox1*, *cox2*, *cox3* |
|  | Complex V (ATP synthase) | *atp1*, *atp4*, *atp6*, *atp8*, |
|  | Cytochrome c biogenesis | *ccmB*, *ccmC*, *ccmFC*, *ccmFN* |
|  | **Translation** | |
|  | Ribosomal proteins (Large subunit, LSU) | *rpl2*, *rpl5*, *rpl6*, *rpl10*, *rpl16* |
|  | Ribosomal proteins (Small subunit, SSU) | *rps1*, *rps2*, *rps3*, *rps4*, *rps7*, *rps10*, *rps11*, *rps12*, *rps13*, *rps14*, *rps19* |
|  | **Others** | |
|  | Intron maturase | *matR*, *mttB* |
|  | | |
| Chloroplast | **Photosynthesis** | |
|  | RuBisCO large subunit | *rbcL* |
|  | Photosystem I | *psaA*, *psaB*, *psaC*, *psaI*, *psaJ* |
|  | Assembly/stability of photosystem I | *ycf3*, *ycf4* |
|  | Photosystem II | *psbA*, *psbB*, *psbC*, *psbD*, *psbE*, *psbF*, *psbH*, *psbI*, *psbJ*, *psbK*, *psbL*, *psbM*, *psbN*, *psbT*, *psbZ* |
|  | Cytochrome b/f complex | *petA*, *petB*, *petD*, *petG*, *petL*, *petN* |
|  | C-type cytochrome | *ccsA* |
|  | ATP synthase | *atpA*, *atpB*, *atpE*, *atpF*, *atpH*, *atpI* |
|  | Nicotinamide adenine dinucleotide dehydrogenase (NADH) | *ndhA*, *ndhB*, *ndhC*, *ndhD*, *ndhE*, *ndhF*, *ndhG*, *ndhH*, *ndhI*, *ndhJ，ndhK* |
|  | **Transcription and translation** | |
|  | Ribonucleic acid (RNA ) polymerase | *rpoA*, *rpoB*, *rpoC1*, *rpoC2* |
|  | Ribosomal protein (Large subunit, LSU) | *rpl2*, *rpl14*, *rpl16*, *rpl20*, *rpl22*, *rpl23*, *rpl32*, *rpl33*, *rpl36* |
|  | Ribosomal proteins (Small subunit, SSU) | *rps2*, *rps3*, *rps4*, *rps7*, *rps8*, *rps11*, *rps12*, *rps14*, *rps15*, *rps16*, *rps18*, *rps19* |
|  | Transfer ribonucleic acid (tRNA) | *trnA*, *trnC*, *trnD*, *trnE*, *trnF*, *trnG*, *trnH*, *trnI*, *trnK*, *trnL*, *trnM*, *trnN*, *trnP*, *trnQ*, *trnR*, *trnS*, *trnT*, *trnV*, *trnW*, *trnY* |
|  | **Others** | |
|  | Maturase | *matK* |
|  | Acetyl-CoA carboxylase subunit | *accD* |
|  | Inorganic carbon uptake | *cemA* |
|  | ATP-dependent protease subunit | *clpP* |
|  | Conserved reading frames (ycfs) | *ycf1*, *ycf2* |
