# Supplementary material for: The Roles of Mitochondrion in Intergenomic Gene Transfer in Plants: A Source and a Pool
Source: Int J Mol Sci. 2018 Feb 11;19(2):547. doi: 10.3390/ijms19020547 (PMC5855769; doi:10.3390/ijms19020547)
Supplement: Supplementary file 1 [file ijms-19-00547-s001.zip › ijms-260413 - Supplementary Figures and Tables/Table S3.pdf]

Table S3. The list of the full names of the analyzed genes and appropriate abbreviations.

Organelles	Category	Genes
Mitochondrion	**Respiration chain complexes**	
	Complex I (Nicotinamide adenine dinucleotide dehydrogenase, NADH)	*nad1*, *nad2*, *nad3*, *nad4*, *nad4L*, *nad5*, *nad6*, *nad7*, *nad9*
	Complex II (Succinate dehydrogenase, SDH)	*sdh3*, *sdh4*
	Complex III (Cytochrome b)	*cob*
	Complex IV (Cytochrome c oxidase)	*cox1*, *cox2*, *cox3*
	Complex V (ATP synthase)	*atp1*, *atp4*, *atp6*, *atp8*,
	Cytochrome c biogenesis	*ccmB*, *ccmC*, *ccmFC*, *ccmFN*
	**Translation**	
	Ribosomal proteins (Large subunit, LSU)	*rpl2*, *rpl5*, *rpl6*, *rpl10*, *rpl16*
	Ribosomal proteins (Small subunit, SSU)	*rps1*, *rps2*, *rps3*, *rps4*, *rps7*, *rps10*, *rps11*, *rps12*, *rps13*, *rps14*, *rps19*
	**Others**	
	Intron maturase	*matR*, *mttB*
Chloroplast	**Photosynthesis**	
	RuBisCO large subunit	*rbcL*
	Photosystem I	*psaA*, *psaB*, *psaC*, *psaI*, *psaJ*
	Assembly/stability of photosystem I	*ycf3*, *ycf4*
	Photosystem II	*psbA*, *psbB*, *psbC*, *psbD*, *psbE*, *psbF*, *psbH*, *psbI*, *psbJ*, *psbK*, *psbL*, *psbM*, *psbN*, *psbT*, *psbZ*
	Cytochrome b/f complex	*petA*, *petB*, *petD*, *petG*, *petL*, *petN*
	C-type cytochrome	*ccsA*
	ATP synthase	*atpA*, *atpB*, *atpE*, *atpF*, *atpH*, *atpI*
	Nicotinamide adenine dinucleotide dehydrogenase (NADH)	*ndhA*, *ndhB*, *ndhC*, *ndhD*, *ndhE*, *ndhF*, *ndhG*, *ndhH*, *ndhI*, *ndhJ*, *ndhK*
	**Transcription and translation**	
	Ribonucleic acid (RNA) polymerase	*rpoA*, *rpoB*, *rpoC1*, *rpoC2*
	Ribosomal protein (Large subunit, LSU)	*rpl2*, *rpl14*, *rpl16*, *rpl20*, *rpl22*, *rpl23*, *rpl32*, *rpl33*, *rpl36*
	Ribosomal proteins (Small subunit, SSU)	*rps2*, *rps3*, *rps4*, *rps7*, *rps8*, *rps11*, *rps12*, *rps14*, *rps15*, *rps16*, *rps18*, *rps19*
	Transfer ribonucleic acid (tRNA)	*trnA*, *trnC*, *trnD*, *trnE*, *trnF*, *trnG*, *trnH*, *trnI*, *trnK*, *trnL*, *trnM*, *trnN*, *trnP*, *trnQ*, *trnR*, *trnS*, *trnT*, *trnV*, *trnW*, *trnY*
	**Others**	
	Maturase	*matK*
	Acetyl-CoA carboxylase subunit	*accD*
	Inorganic carbon uptake	*cemA*
	ATP-dependent protease subunit	*clpP*
	Conserved reading frames (ycfs)	*ycf1*, *ycf2*
